# Experiments in the Use of Tin for Insertion of Teeth

**Published:** 1850-10

**Authors:** W. A. Royce

**Affiliations:** Dental Surgeon, of Newburgh, N. Y.


### ARTICLE II.

*Experiments in the Use of Tin for Insertion of Teeth.* By W. A. Royce, Dental Surgeon, of Newburgh, N. Y.

My first effort was made in 1836, for a lady aged about sixty, who had lost her inferior incisors and some adjacent teeth, in a sound and beautiful state, by harsh brushing, and desired me to replace them with little or no expense.

After an impression and plaster cast were obtained, the ejected teeth were cut off near the end of the root, and perforated with a small drill, and strung upon a silver wire; then placed on the plaster cast, and supported by the wire around the plaster teeth, in proper position, and at such space above the cast of gum as would permit molten tin to flow beneath the teeth; after which a curb was constructed around the teeth and gum, properly dried, and the tin poured in, thus forming a base fitting the gum and clasps to the permanent teeth, and holding the inserted teeth firmly. Filed, polished and introduced, it gave great satisfaction until the death of the patient, which occurred some eight or ten years thereafter.

My second use of tin was in 1838, for a lady aged about forty—to insert her own incisors, which had fallen out, and afford support to an entire upper set by spiral springs—tin base to rest upon gum and badly worn down teeth, and attach to a few loose, attenuated teeth—a temporary resort for a patient imbued with more fear than discretion.

The tin base enclosing the teeth was stereotyped over the gum and teeth, as in first case, and filed, fitted, polished and springs attached.

Calling some two or three weeks thereafter, she said it had caused her much pain, but was then comfortable; and it was a long time in service, with about the usual results. I became prejudiced, however, against tin to lay on the gum, and used but one more, without plate, to lay on the gum. This third case was a tin base, in an entire upper and lower case, for a gentleman about sixty-five, whose saliva was so inert that sixteen carat gold plate would not tarnish sooner than eighteen carat usually does. He wore, and used severely, his teeth for about two years, when I struck and fitted a gold plate, of about No. 30, and stereotyped the tin on it—embracing the teeth which had been freed from their former base for this melting. An accident occurred in this experiment: the tin used was too hot, and amalgamated with the gold plate just where the current of molten tin impinged on it. Thus I learned that tin should not be hotter than barely to produce limpidity. I think I did not misjudge when, after several years use of these cases, I abandoned the employment of tin for the part coming in contact with the gum. It did not produce irritation on the central part of the gum; but toward either outer or inner border, in contact with integuments, often abundant, and sensitive, it seemed to me to be more irritating. Tin will corrode in contact with excoriated surfaces in the mouth, and throw up a slight pile of grey (chloride?) compound, insoluble in the saliva, somewhat harsh to the feel, crisp to the file or scraper, and at least mechanically irritating to the excoriated surfaces which caused it.

My fourth experiment was for an elderly lady—mouth totally toothless; desired the lower denture only, on a very broad inferior maxilla and large alveolar ridge, to match the teeth in a small superior maxilla and gum. This was another nothing or next to nothing pay case; but I attempted to better my former practice, and struck and fitted a silver plate. Set French teeth with silver wire studs soldered to plate; stereotyped the tin gum on the plate; gilded all parts finely and prettily. Thought I had made a fine advance. It was tried fairly for several days, and proved to be a superior sialagogue. I question the superiority of mercury to produce ptyalism.

My fifth effort was to insert an entire upper and lower denture; lower gum narrow, and summit thin. The teeth were mounted in the usual way on gold plate, except the metallic gum was tinman's soft solder, flowed around the teeth with a tinker's iron. Twelve or eighteen months use showed the gum shelling off—a black and separating mass, like the moss on the bark of a forest tree—another mortifying experiment, but easily remedied by substituting pure tin for tinker's solder.

The sixth experiment was the same as the fifth, except the lower gum was wider, and silver linings were used, and the tin made to serve both to fasten the teeth and load the plate. The silver linings evinced galvanic polarity, causing frequent pricking shocks to the tongue. Gold lined teeth were substituted, and the shocks obviated This experiment, made in 1841, has decided all my practice since. I get up my lower cases, when entire, about the thickness known as No. 30. Strike the plate about one-sixteenth to one-eighth inch extra wide; curve up the edge the extra width on the outer border; coat the upper surface with pure tin, using a tinker's soldering copper, and saturated muriate of zinc as flux. The teeth are next arranged so as to articulate properly, but if admissible their lower ends are broken off, to permit a considerable body of tin to flow under them; line them with the same thickness of gold plate; place them in position, with wax on their lined faces; set them in plaster, and when the wax can be removed, proceed to solder them by their linings, with tin, to the plate, filling all smoothly from tip of teeth to lower edge of gold plate; remove the plaster on the outside of the teeth, and fill up the interstices and base of teeth in such quantity as pleases; file off any excess of tin any where; preserve a thick smooth border to the plate, both within and without the arch; scrape down all asperities not to be reached by the file: mat the surface with fine sandpaper; clean out the sand and burnish smoothly; then finish beautifully with flannel and chalk, which last will ever after preserve the cases cleanly, if used daily. By this method any or all the teeth are readily detached, set in new position, and re-secured, ad infinitum. For fractional cases, gold plate should be heavier, and the clasps soldered on with gold solder, as in using the tin solder they become detached in use, or when repaired or altered.

Very extensive accommodations can also be afforded to the gum, by taking the tin gum or teeth off over the misfitting section, bending the plate to fit, and again restoring the gum or teeth.

To stereotype the gum, instead of the process of flowing it on with a soldering copper, I have tinned the linings after riveting—set the teeth in position—placed pure yellow wax around the teeth, just as I wished the tin to be added—cast the whole in plaster—exposed the whole to such heat as would dry the plaster and cause the wax to be absorbed by it—cut an opening to pour the tin through before such drying; take a proper quantity of tin and fuse it in an iron spoon; watch the fusion, and permit no greater heat than sufficient to cause the convex edges which first are prominent around the edges, to disappear—in other words, to make the whole surface of the molten tin flat; then pour it in the plaster flask, and soon as it has cooled break off the plaster, when the tin will be found to occupy precisely, or very nearly so, the exact place of the wax. Little sand-papering is generally all the trimming needed; sometimes certain spots may require a touch of the soldering copper.

The immunity against corrosion that cadmium possesses, seems to indicate that it might be better than tin. I have not tried it. My efforts to coat tin with galvanic gilding were futile, as the gold very soon disappeared from the friction of aliment.

